# Bacterial Compatibility/Toxicity of Biogenic Silica (b-SiO_2_) Nanoparticles Synthesized from Biomass Rice Husk Ash

**DOI:** 10.3390/nano9101440

**Published:** 2019-10-11

**Authors:** Sanjeev K. Sharma, Ashish R. Sharma, Sudheer D. V. N. Pamidimarri, Jyotshana Gaur, Beer Pal Singh, Sankar Sekar, Deuk Young Kim, Sang Soo Lee

**Affiliations:** 1 Department of Physics, C.C.S. University, Meerut Campus, Meerut UP 250004, India; sanjeevlrs73@gmail.com (S.K.S.); jyotshanaphysics@gmail.com (J.G.); drbeerpal@gmail.com (B.P.S.); 2 Institute for Skeletal Ageing & Orthopedic Surgery, Hallym University-Chuncheon Sacred Heart Hospital, Chuncheon, Gangwon-Do 24253, Korea; boneresearch@hallym.ac.kr; 3 Division of Physics and Semiconductor Science, Dongguk University-Seoul, Seoul 04620, Korea; 4 Institute of Biotechnology, Amity University, Raipur, Chhattisgarh 493225, India; pdvnsudheer@gmail.com

**Keywords:** biogenic silica (b-SiO_2_), microstructural analysis, bacterial compatibility, anti-bacterial, *E. coli*, *S. aureus*

## Abstract

Biogenic silica (b-SiO_2_) nanopowders from rice husk ash (RHA) were prepared by chemical method and their bacterial compatibility/toxicity was analyzed. The X-ray diffractometry (XRD) patterns of the b-SiO_2_ nanopowders indicated an amorphous feature due to the absence of any sharp peaks. Micrographs of the b-SiO_2_ revealed that sticky RHA synthesized SiO_2_ nanopowder (S1) had clustered spherical nanoparticles (70 nm diameter), while b-SiO_2_ nanopowder synthesized from red RHA (S2) and b-SiO_2_ nanopowder synthesized from brown RHA (S3) were purely spherical (20 nm and 10 nm diameter, respectively). Compared to the S1 (11.36 m^2^g^−1^) and S2 (234.93 m^2^g^−1^) nanopowders, the S3 nanopowders showed the highest surface area (280.16 m^2^g^−1^) due to the small particle size and high porosity. The core level of the X-ray photoelectron spectroscopy (XPS) spectra showed that Si was constituted by two components, Si 2p (102.2 eV) and Si 2s (153.8 eV), while Oxygen 1s was observed at 531.8 eV, confirming the formation of SiO_2_. The anti-bacterial activity of the b-SiO_2_ nanopowders was investigated using both gram-positive (*Escherichia coli*) and gram-negative (*Staphylococcus aureus*) microorganisms. Compared to S2 and S3 silica nanopowders, S1 demonstrated enhanced antibacterial activity. This study signifies the medical, biomedical, clinical, and biological importance and application of RHA-mediated synthesized b-SiO_2_.

## 1. Introduction

The most economic agro-waste material, rice husk (RH), a rice by-product, is abundantly available for the synthesis of biogenic silica from rice husk ash (RHA) [1]. Silica (SiO_2_) has already been utilized in the fields of electronics and medical treatment [2,3]. Silica can also be synthesized from a number of natural resources such as mollusks, sponges [4], protozoa [5], quartz sands, and higher plants [6]. Biogenic silica can be synthesized from rice by-products at a rate of gigatons/year [7,8]. RH is one of the most environmentally friendly materials for biogenic silica. RH contains a chemical composition (in percentages) of organic components, such as cellulose (~38%), hemicellulose (~20%), and lignin (~22%) (~80%), and inorganic constituents (SiO_2_) (~20%) [1,9,10]. Currently, RH is exploited in different applications, such as fuel for boilers, electricity generation, feedstock for biofuel production, and as a bulking agent for composting animal manure. The utilization of RH and RHA to prepare biogenic silica (b-SiO_2_) will lead to new commercial applications. b-SiO_2_ nanopowders can be synthesized from RHA via various methods, including chemical methods (acid/alkali leaching and post-heat treatment) [11,12,13,14], combustion synthesis [15], microwave hydrothermal synthesis [16], fluidized bed and flame synthesis [17,18], pressurized hot-water treatment [19], precipitation [20], and non-isothermal methods [21]. Among these approaches, the chemical method is a simple and successful technique for synthesizing b-SiO_2_ nanopowders from RHA [14,22].

Capeletti et al. [23] demonstrated that tailored silica nanoparticles possess antibacterial properties with reduced cellular toxicity against cell lines. In addition, biogenic SiO_2_ nanoparticles also tend to show biocompatibility with human cell lines [7]. Alshatwi et al. [24] successfully demonstrated the biocompatible nature of b-SiO_2_ nanoparticles. The extraction of b-SiO_2_ nanoparticles from RHA improves the understanding of their characteristics. According to the literature, the effect of the crystallite size and surface area of b-SiO_2_ nanopowders synthesized from sticky RHA, red RHA, and brown RHA and their bacterial compatibility/toxicity with particular gram-positive (*Staphylococcus aureus*) and gram-negative bacteria (*Escherichia coli*) have not yet been studied. The microstructure and bacterial compatibility/toxicity of b-SiO_2_ is expected to vary with the geographical provenance, type of RHA, and the size of nanoparticle. Uniformly distributed spherical b-SiO_2_ nanopowders have not been well matched for the requirements of future prospective applications.

The objective of this work was to synthesize b-SiO_2_ nanoparticles from different RHAs and test their bacterial compatibility/toxicity. The microstructural, elemental, functionality, porosity, and binding energy characteristics of b-SiO_2_ were evaluated using X-ray diffractometry (XRD), field emission scanning electron microscopy (FE-SEM), high-resolution transmission electron microscopy (HR-TEM), surface area electron diffraction (SAED), Brunauer–Emmett–Teller (BET) analysis, X-ray photoelectron spectroscopy (XPS), and Raman spectroscopy. An examination of the microbial inhibition was performed on two different bacterial species: *E. coli* (gram-negative) and *S. aureus* (gram-positive).

## 2. Materials and Methods

### 2.1. Materials

All of the chemicals and solvents were purchased from Merck and Sigma Aldrich (St. Louis, MO, USA), were of AR grade, and were used without further purification. Brown RHA and red RHA were collected from Tamil Nadu, India, while sticky RHA was obtained from South Korea.

### 2.2. Synthesis of b-SiO_2_ Nanopowder

The b-SiO_2_ nanopowders were synthesized from various RHAs using a simple acid pre-treatment method as discussed previously by Sankar et al. [25]. The three types of RHs (sticky, red, and brown) were burned in an open environment and their ashes were collected. A total of 3.0 g of each RHA was first stirred with 45 mL of 10% HCl for 2 h to remove the metal ions inside and denoted as leached RHA (L-RHA). The L-RHA was sonicated for 50 min at a power of 30 W and a frequency of 20 k Hz. The sonicated L-RHA was denoted as sonicated leached RHA (SL-RHA). The SL-RHA was filtered and washed with a large amount of DI water and dried at 150 °C for 24 h in a conventional electric oven. The dry powders obtained were transferred from a petri dish to an alumina crucible and then annealed at 700 °C with a ramp rate of 5 °C min^−1^ in a muffle furnace at atmospheric conditions for 2 h. Finally, white colored mesoporous b-SiO_2_ nanopowders were obtained from all three RHAs denoted as sticky RHA: S1, red RHA: S2, and brown RHA: S3, respectively.

### 2.3. Characterization of the b-SiO_2_ Nanopowders: SEM, TEM, XRD, BET, XPS, EDAX, and Raman Spectroscopy

The surface morphology and grain sizes of the synthesized S1, S2, and S3 b-SiO_2_ nanopowders were examined using FE-SEM (Hitachi, S-4800) and HR-TEM. The purity and elemental composition of the sample was identified by analyzing the atomic percentage of silica using Energy Dispersive X-Ray Analysis (EDAX) (Bruker Inc., Madison, WI, USA). The crystallinity of the synthesized b-SiO_2_ nanopowders was investigated via X-ray diffractometry (XRD) using a CuKα1 radiation source (λ = 1.5405 Å) under a constant current of 40 mA at 40 kV and with a diffraction angle (2θ) scan range of 5°–80°. The absence of any sharp peaks in the XRD patterns confirmed the amorphous nature of the materials and further justified the selected area electron diffraction (SAED) pattern area. Adsorption-desorption isotherms (BET) were used to study the S1, S2, and S3 surface areas. The chemical bonding states were determined from X-ray photoelectron spectroscopy (XPS) (Thermo Fischer Scientific) by an excitation radiation source of monochromatic Al-Kα (1486.6 eV). The charging of the samples was corrected by setting the binding energy of the adventitious carbon (C1s) at 284.5 eV. The analysis was conducted at ambient temperature at a pressure of typically less than 10-6 Pa. Before recording the data, all of the samples were degassed in a vacuum overnight. The Raman spectra of the S1, S2, and S3 nanopowders were performed using a (LabRAM Jobin Yvon HR 800 UV, Kyoto, Japan) spectrometer over a wave number range of 100–1200 cm^−1^ at room temperature.

### 2.4. Bacterial Compatibility/Toxicity Evaluation

Bacteriological tests were conducted using two different pathogenic species of gram-negative and positive bacterial strains. Gram-negative *E. coli* (ATCC 25922) and gram-positive *S. aureus* (ATCC 29213) strains were cultivated overnight in a Luria–Bertani (LB) nutrient medium at 37 °C. The culture was sub-cultured in fresh LB medium using 2% inoculum and grown to the mid-log phase (Optical Density of 0.6 at 660 nm). The cultures were centrifuged, washed with saline solution (0.85% NaCl), and suspended using saline. The S1, S2, and S3 nanoparticles at concentrations of 0.1 mg mL^−1^ were loaded onto a culture plate. A control sample was maintained without the nanoparticle treatment and proceeded parallel to the experiment to assess the colony-forming unit (CFU) changes with the experiment to ensure that the decline in the CFU was due to the material’s toxicity. Compared to the control, the CFU changes were calculated at intervals of 90 min for both *E. coli* and *S. aureus*. At each interval, the samples were serially diluted and 100 µL of each dilution was plated in plain LB agar. The CFU per mL from each interval aliquot was calculated from the appropriate dilution via respective colony counting. The same procedure was used to determine the bacterial count followed by sonication to assess the sample behavior.

### 2.5. Quantification of the Adhered Bacteria to the b-SiO_2_ Nanopowders

The bacterial adherence was studied using a protocol adopted from Kinnari et al. [26]. The samples after treatment with the bacterial culture grown in the mid-log phase (OD of 0.6 at 660 nm) were prepared as described in Section 2.4. After 7.5 h of treatment, each material was washed with saline three times to remove the free bacterial culture. The samples were placed in an equal amount of saline (0.1 mg mL^−1^). The sonication was conducted in a low-power (150 W) ultrasonic bath (Sonorex Super, Bandelin, Berlin, Germany) for 5 min. After sonication, the liquid supernatants underwent CFU determination as mentioned in the previous section. Following the sonication of the materials, they were washed three times with saline to remove the dislodged bacterial culture. The material was then streaked in the LB agar plates to find any bacterial cells that adhered to the material.

## 3. Results and Discussion

**Figure 1:**
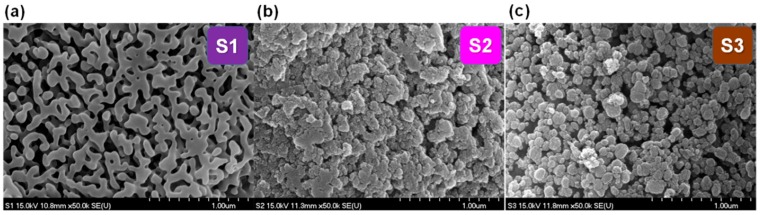
Images of the synthesized biogenic silica, (**a**) S1, (**b**) S2, and (**c**) S3 nanopowders.

Figure 1a–c shows FE-SEM images of the S1, S2, and S3 b-SiO_2_ nanopowders synthesized from the sticky RHA, red RHA, and brown RHA, respectively. The S1 microstructure had cluster-type spherical nanoparticles synthesized from the sticky RHA. The other two b-SiO_2_ nanopowders (S2 and S3) synthesized from the red RHA and brown RHA had uniform spherical nanoparticles with a narrow size distribution. When the RHA changed from sticky to brown, the particle size of the b-SiO_2_ nanopowders decreased from 70 to 10 nm.

The XRD patterns of the S1, S2, and S3 b-SiO_2_ nanopowders confirmed the amorphous nature of the materials (Supplementary Figure S1). The prepared S1, S2, and S3 nanopowders showed a broad intense peak at 2θ = 22°, indicating the presence of silica nanoparticles. No other impurities were detected even when the materials were characterized in triplicate. The absence of sharp peaks in the S1, S2, and S3 XRD patterns confirmed the amorphous nature of the material. Previous studies proved that RH-derived silica nanoparticles have an amorphous structure [14,24,27].

**Figure 2:**
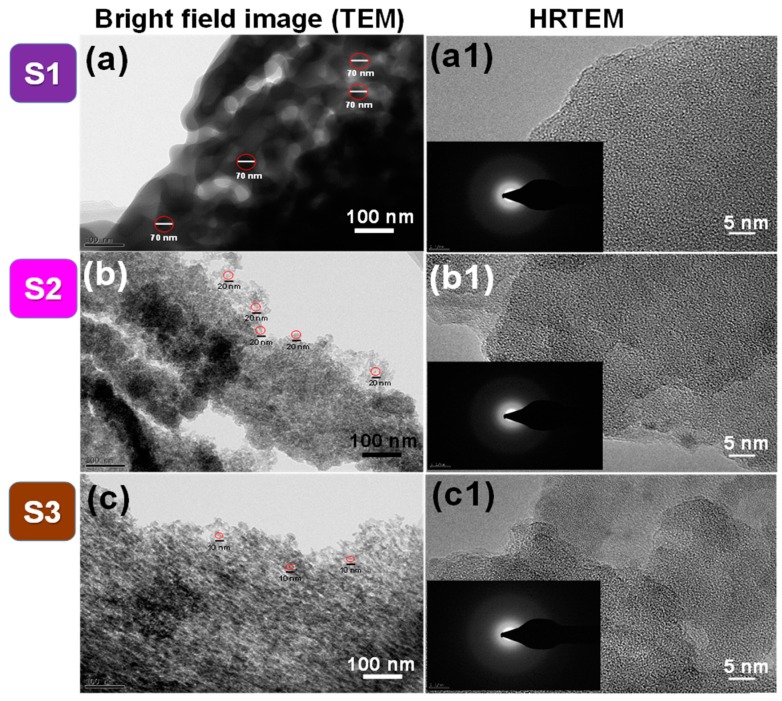
Analysis of S1, S2, and S3 nanopowders: (**a**–**c**) bright field transmission electron microscopy (TEM) images; (**a1**–**c1**) high resolution transmission electron microscopy (HR-TEM) images; the surface area electron diffraction (SAED) pattern of b-SiO_2_ nanopowders are inset in figures of HR-TEM images.

Bright field TEM images of S1, S2, and S3 are shown in Figure 2a–c, respectively. The S1 nanopowder with cluster-type nanoparticles had irregular geometry with a wide size distribution of spherical shapes of approximately 70 nm. Every primary particle was interconnected and adhered to each other. The S2 and S3 nanopowders clearly showed spherical nanoparticles with narrow size distributions in agglomerated species. The average crystal size of the S2 and S3 nanopowders was 20 nm and 10 nm, respectively. Compared to the crystal size of the S1, S2, and S3 nanopowders, the S3 nanopowder had the smallest spherical size with a uniform narrow size distribution. Figure 2a1–c1 show HR-TEM images of the S1, S2, and S3 nanopowders, respectively. The absence of uniform and periodic lattice spacing in the HR-TEM images also confirmed the amorphous nature of the b-SiO_2_ nanopowders. Selected area electron diffraction (SAED) patterns of the S1, S2, and S3 nanopowders are shown in the insets in Figure 2a1–c1, respectively. The electron diffraction rings of the S1, S2, and S3 nanopowders also indicated the amorphous phase of the silica nanoparticles. The EDAX analysis confirmed the purity of silica in the given sample (Supplementary Figure S2).

**Figure 3:**
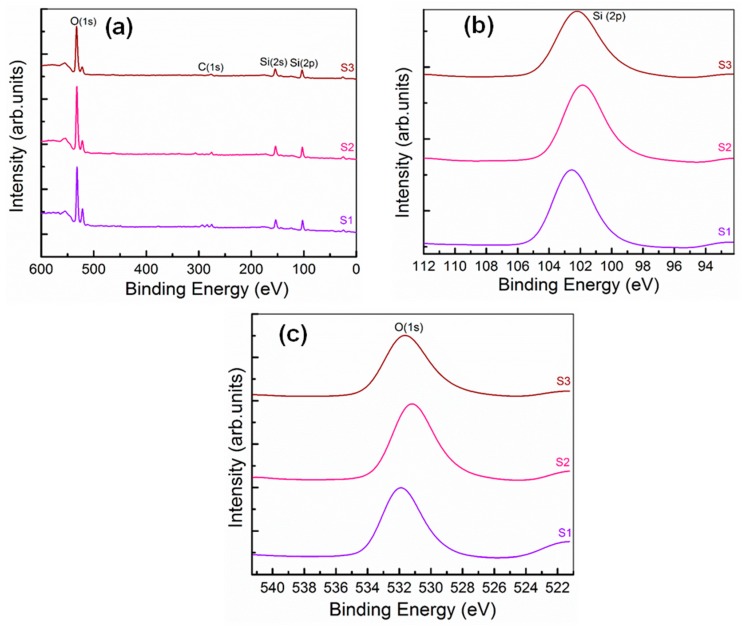
X-ray photoelectron spectroscopy (XPS)-spectra of S1, S2, and S3 nanopowders, (**a**) full scale spectra, (**b**) the core level spectra of Si (2p), and (**c**) the core level spectra of O (1s).

The chemical bonding of the b-SiO_2_ nanopowders was analyzed via XPS. The full XPS survey spectra of the S1, S2, and S3 nanopowders are shown in Figure 3a. The core level spectra of Si(2p) and O(1s) indicating the presence of silicon and oxygen components of b-SiO_2_ are shown in Figure 3b. The S1, S2, and S3 nanopowders showed strong Si(2p) peaks at 102.6, 101.8, and 102.2 eV, respectively, demonstrating the presence of pure b-SiO_2_. An absorbed Si(2s) peak was observed at 153.8 eV, indicating the presence of Si species in the samples. O1s peaks of the b-SiO_2_ nanopowders were observed at 531.8, 531.1, and 531.5 eV, demonstrating the contribution of oxygen in the silica. The presence of C content in the XPS spectra was also observed at 284.5 eV due to the charging of the samples [28,29,30,31]. The binding energy of the Si and O in the b-SiO_2_ nanopowders varied only slightly due to the type of RHA. The observed XPS results also supported the energy-dispersive X-ray spectroscopy characteristics. The surface areas of the b-SiO_2_ nanopowders were determined using the Brunauer–Emmett–Teller (BET) method. The detail of adsorption–desorption isotherm measurements of the S1, S2, and S3 b-SiO_2_ nanopowders are given in the supplementary information (Supplementary Figure S3). The specific surface areas of the S1, S2, and S3 b-SiO_2_ nanopowders were 11.36, 234.93, and 280.16 m2 g^−1^, respectively. The highest surface area was in the biogenic silica synthesised brown RHA and was found in the category IV model (H2-type hysteresis loop) that justified International Union of Pure and Applied Chemistry (IUPAC) classification [21,32].

**Figure 4:**
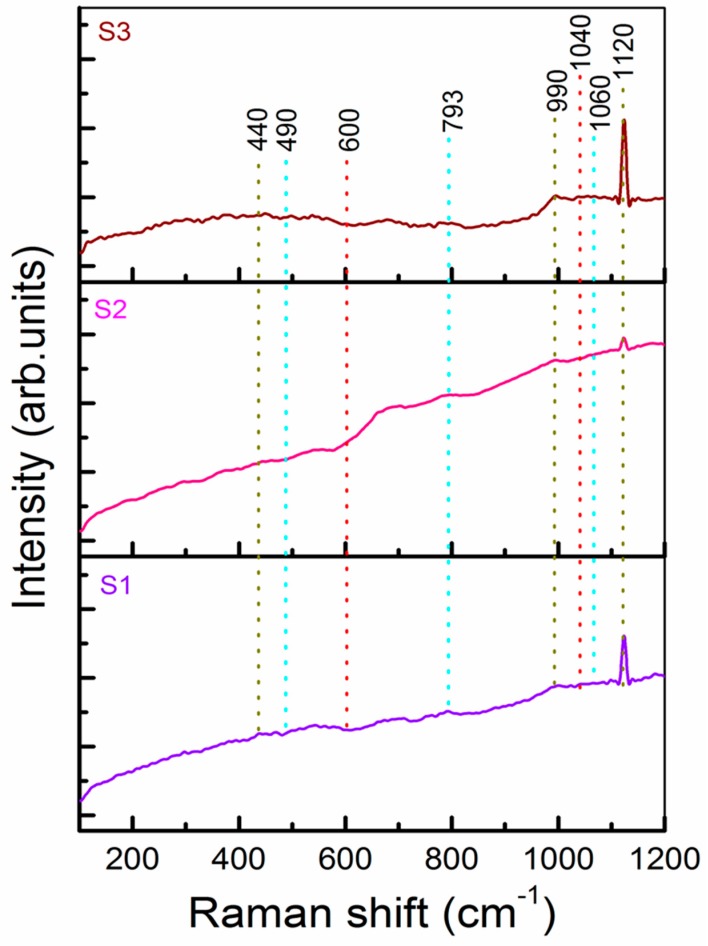
Spectra of S1, S2, and S3 nanopowders.

Figure 4 shows the Raman spectra of the S1, S2, and S3 b-SiO_2_ nanopowders in a wavelength range of 100–200 cm^−1^. Various bands were observed in the spectra such as a bending mode at 440 cm^−1^ and a stretching mode at 793, 1040, and 1120 cm^−1^. These Raman spectra bands represented the siloxane bond (Si-O-Si) in the intrinsic silica structure [33,34]. A small band at approximately 440 cm^−1^ indicated the symmetric stretching ~1 mode of the SiO_2_. The symmetric stretching bands at 490 (D1 peak) and 793 (D2 peak) cm^−1^ exhibited the bulk and internal surface defects of the fourfold and threefold rings of the SiO_2_ tetrahedral structure, respectively [35,36]. The less intense bands located at 990 cm^−1^ were related to the Si-OH stretching modes of the free and hydrogen-bonded SiOH groups. The peaks located at 1060 and 1120 cm^−1^ indicated the presence of transverse optical (TO) and longitudinal optical (LO) pairs, respectively [33]. These results clearly indicate that the synthesized b-SiO_2_ nanopowders contained highly pure SiO_2_ components.

**Figure 5:**
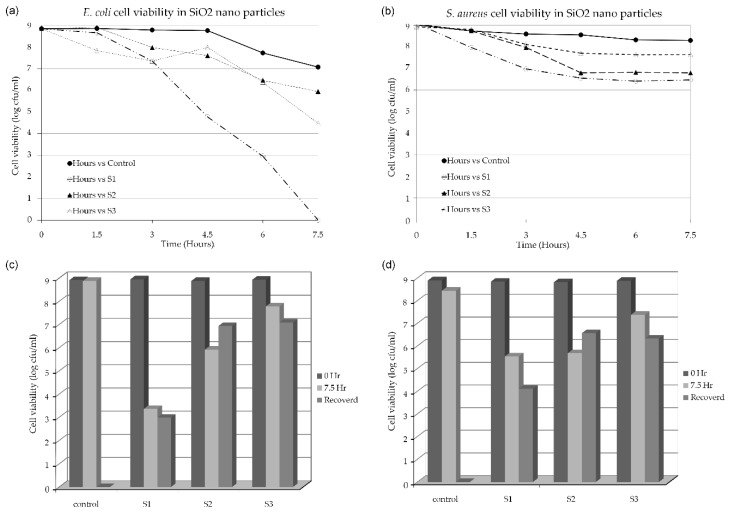
Bacterial cell viability under the treatment of S1, S2, and S3 nanopowders compared with control (without treatment); (**a**) *E. coli* and (**b**) *S. aureus*. Viable cell count after the treatment of culture for 7.5 h by S1, S2, and S3 nanopowders recovered via sonication and comparison with control (without treatment); (**c**) *E. coli* and (**d**) *S. aureus*.

**Figure 6:**
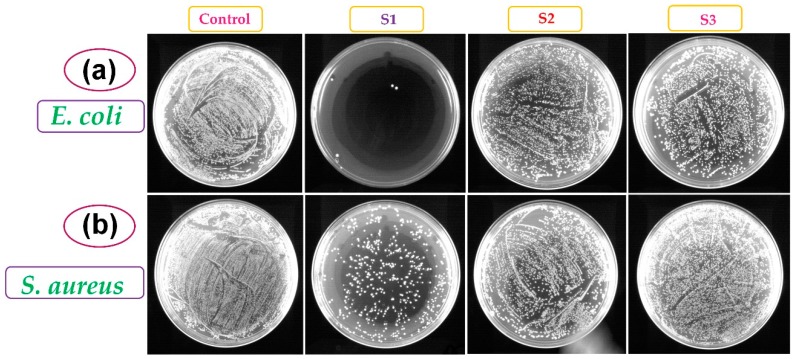
Culture plates exposed with 0.1 mg biogenic silica, S1, S2, and S3 nanopowders for 7.5 h were plated on Luria–Bertani (LB) agar plates (10-4 dilution); (**a**) *E. coli* and (**b**) *S. aureus*.

The bacteriological toxicity of the S1, S2, and S3 b-SiO_2_ nanopowders was evaluated using *E. coli* and *S. aureus* as representative organisms of gram-negative and gram-positive bacteria. Our result demonstrated that there was a decline in the bacterial count of all three materials checked in triplicate (Figure 5 and Figure 6). In the *E. coli*, the S1 nanopowder had a complete decline in the viable cell count after 7.5 h of treatment, while S2 and S3 nanopowders did not show the complete elimination of the bacterial cell viability. However, the complete decline of *S. aureus* was not observed in any of the b-SiO_2_ nanopowders. The population of *S. aureus* was lower for the initial 4.5 h and then the bacterial population did not decrease (*S. aureus*) in the three b-SiO_2_ nanopowders. Therefore, the b-SiO_2_ nanopowders demonstrated adhesion properties toward the bacterial cells. For further investigation, we evaluated the bacterial adhesion on the material surfaces and assessed if the cells physically adhered to the material and the actual decline in the cell count due to the bacterial toxicity of the materials. To evaluate our results, we followed a study by Kinnari et al. [26], in which the cells were dislodged by mild sonication, which did not damage the cells. After 7.5 h of treatment, the material was separated from the suspended cells. The cells were washed with saline and mild sonication was performed (the conditions were optimized to not affect the cell viability). The results showed a significant cell count recovered by mild sonication (Figure 5c,d). The data clearly demonstrated that all three b-SiO_2_ nanopowders synthesized from different RHAs did not have similar properties with the biological cells. S2 and S3 showed significant recovery, while S1 did not. The negligible cell count recovered from the S1 material was likely due to the strong adhesion that resulted in no recovery via sonication. To assess this, the remaining material was streaked in a plate containing solid LB agar media. The S2 and S3 nanopowders demonstrated culture growth in the *E. coli* and *S. aureus* plates, but S1 showed no culture growth (Supplementary Figure S4a,b). The results clearly indicated that the S1 nanopowder had significant bacterial toxicity, while the S2 and S3 nanopowders had little or no toxicity toward the bacterial cells. The b-SiO_2_ nanopowders had significant adhesion to the *E. coli* and *S. aureus* cells. It is plausible that the high anti-bacterial activity of S1 is due to its high adhesion ability on the bacterial cell wall. In addition, the high anti-bacterial activity of S1 towards *E. coli* is due to the differences in the cell wall compositions of gram positive (*S. aureus*) and gram negative (*E. coli*) bacterial cells. Moreover, the bacterial toxicity of the b-SiO_2_ nanopowders might also vary with respect to the variation in the RHAs and/or the microstructure depending on the geographical provenance. The results revealed that the S1 nanopowder has high anti-bacterial property and high bio-adhesion, while the S3 nanopowder had higher bacterial compatibility than the S1 and S2 nanopowders.

## 4. Conclusions

b-SiO_2_ nanopowders were successfully synthesized from different RHAs using the chemical method. SEM and TEM micrographs of the b-SiO_2_ nanopowders affirmed the spherical shape and a uniform size distribution. The elemental composition, chemical bonding analysis, and binding energy of the b-SiO_2_ nanopowders confirmed the pure silica phase. The absence of sharp peaks in the XRD patterns and the electron diffraction rings in the SAED patterns demonstrated the amorphous nature of the materials. The antibacterial and adhesion characteristics of the b-SiO_2_ (S3) nanopowders showed excellent bacterial compatibility with *E. coli* and *S. aureus* bacterial strains and led to a conclusion of environmental biota. The S3 nanopowder synthesized from brown RHA had the smallest particle size, highest surface area, and superior bacterial compatibility and can be considered the most compatible material for biomedical applications.

## Supplementary Materials

The following are available online at https://www.mdpi.com/2079-4991/9/10/1440/s1, Figure S1: XRD, Figure S2: EDAX spectra, Figure S3: BET surface analysis, Figure S4: After sonication the bacterial growth of *E. coli* and *S. aureus.*
