# Supplementary material for: Bacterial Compatibility/Toxicity of Biogenic Silica (b-SiO2) Nanoparticles Synthesized from Biomass Rice Husk Ash
Source: Nanomaterials (Basel). 2019 Oct 11;9(10):1440. doi: 10.3390/nano9101440 (PMC6835479; doi:10.3390/nano9101440)

*Article*

# Bacterial Compatibility/Toxicity of Biogenic Silica (b-$SiO_2$) Nanoparticles Synthesized from Biomass Rice Husk Ash

**Sanjeev K. Sharma [1,2,3], Ashish R. Sharma [2], Sudheer D. V. N. Pamidimarri [4], Jyotshana Gaur [1], Beer Pal Singh [1], Sankar Sekar [3], Deuk Young Kim [3,*] and Sang Soo Lee [2,*]**

[1] Department of Physics, C.C.S. University, Meerut Campus, Meerut UP 250004, India; sanjeevlrs73@gmail.com (S.K.S.); jyotshanaphysics@gmail.com (J.G.); drbeerpal@gmail.com (B.P.S.)

[2] Institute for Skeletal Ageing & Orthopedic Surgery, Hallym University-Chuncheon Sacred Heart Hospital, Chuncheon, Gangwon-Do 24253, Korea; boneresearch@hallym.ac.kr

[3] Division of Physics and Semiconductor Science, Dongguk University-Seoul, Seoul 04620, Korea; sanssekar@gmail.com

[4] Institute of Biotechnology, Amity University, Raipur, Chhattisgarh 493225, India; pdvnsudheer@gmail.com

* Correspondence: dykim@dongguk.edu (D.Y.K.); 123sslee@gmail.com (S.S.L.)

Received: 28 July 2019; Accepted: 2 October 2019; Published: 11 October 2019

**Figure S1: XRD**

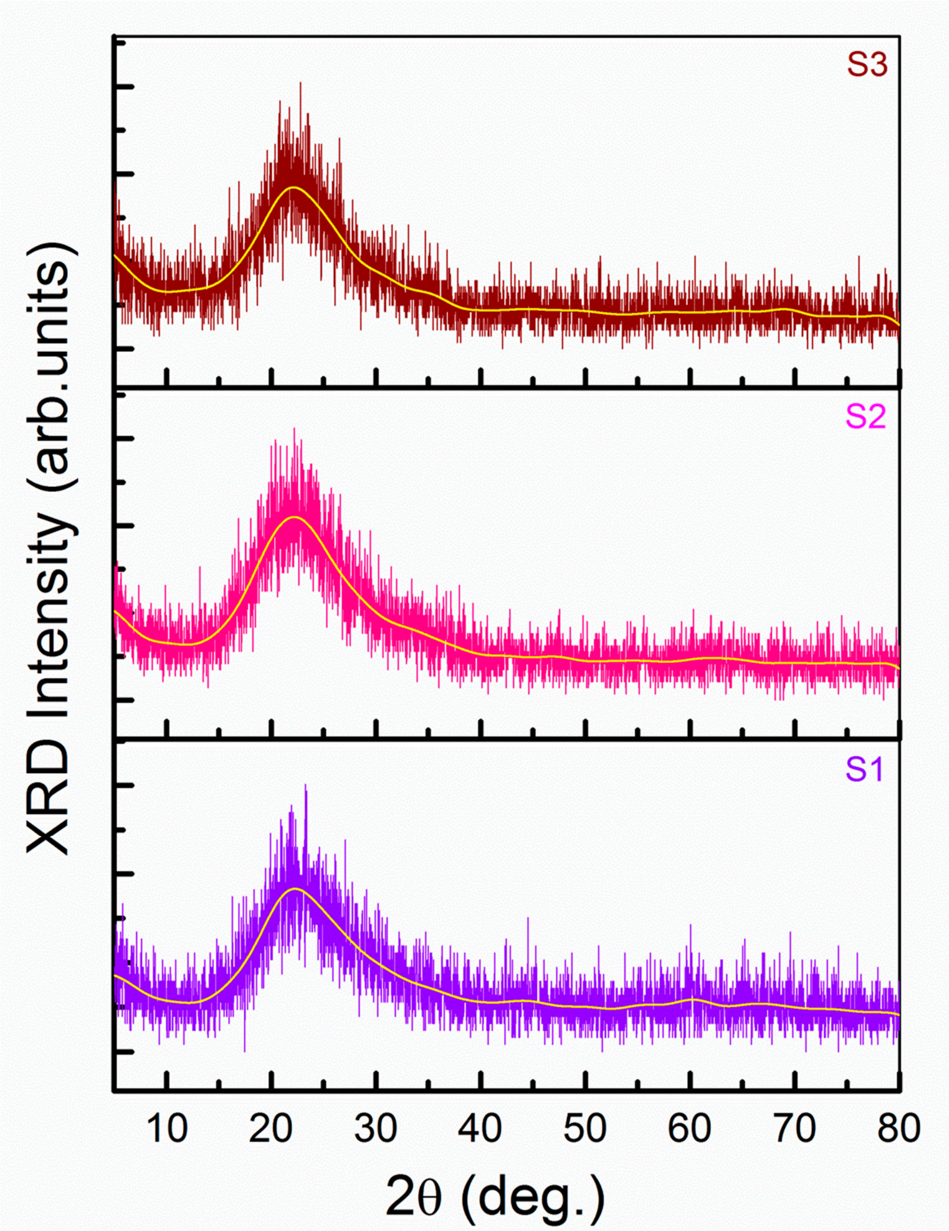

**Figure S2: EDAX spectra**

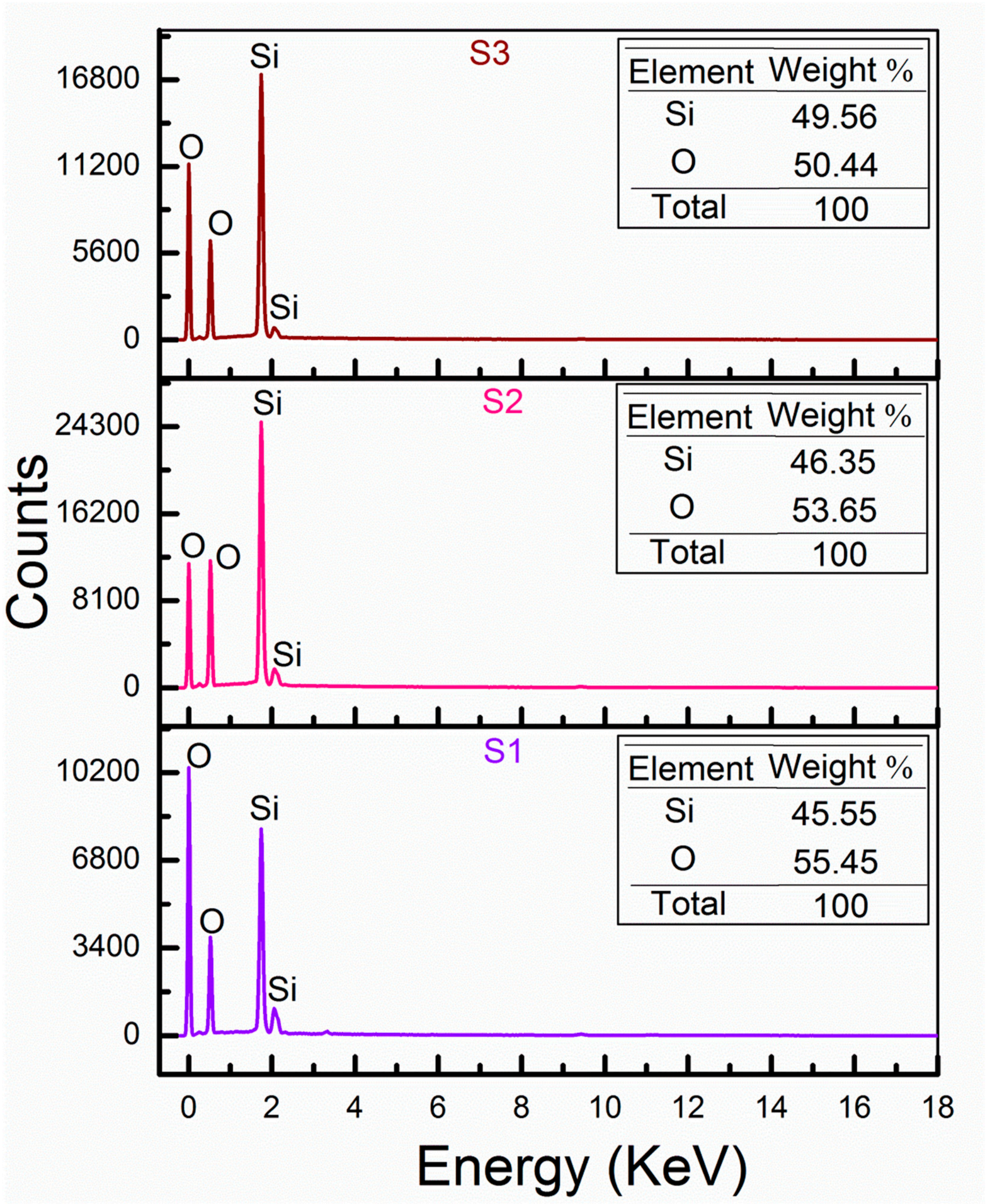

**Figure S3: BET surface analysis**

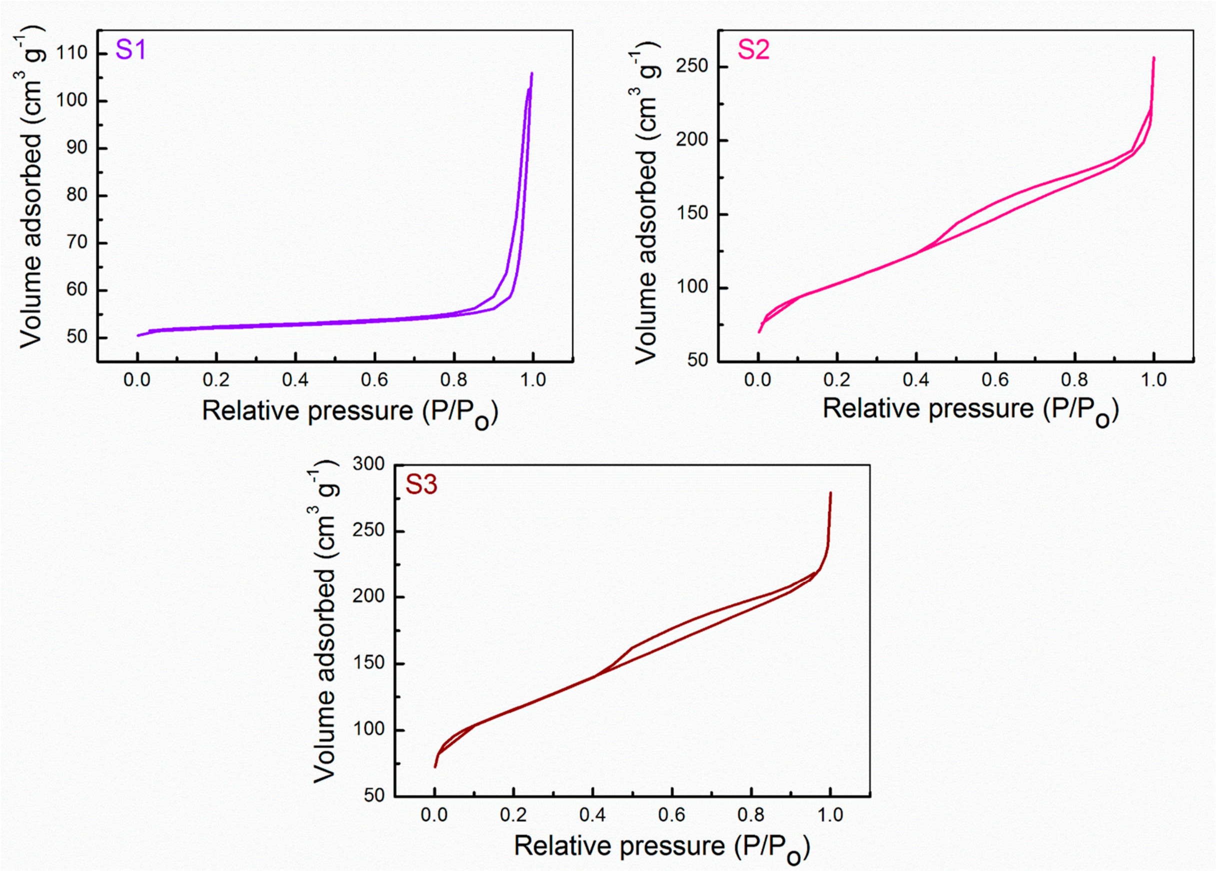

**Figure S4: After sonication the bacterial growth of E.coli and S.aureus**

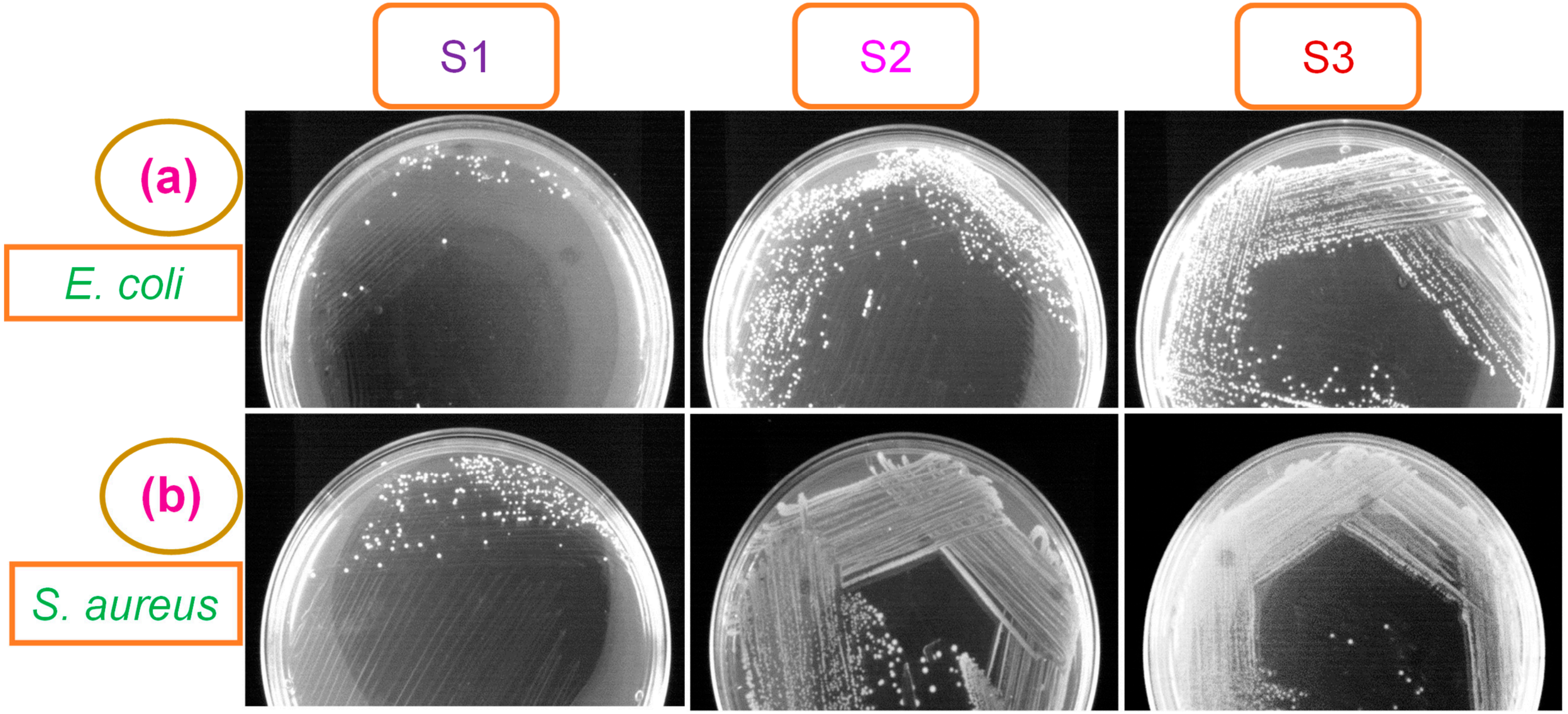

Supplement: Supplementary file 1 [file nanomaterials-09-01440-s001.pdf]
